# Registration of systematic reviews in PROSPERO: 30,000 records and counting

**DOI:** 10.1186/s13643-018-0699-4

**Published:** 2018-02-20

**Authors:** Matthew J. Page, Larissa Shamseer, Andrea C. Tricco

**Affiliations:** 1 School of Public Health and Preventive Medicine, Monash University, 553 St Kilda Road, Melbourne, VIC 3004 Australia; 2 Centre for Journalology, Clinical Epidemiology Program, Ottawa Hospital Research Institute, Ottawa, K1H 8L6 Canada; 3 School of Epidemiology and Public Health, Faculty of Medicine, University of Ottawa, Ottawa, K1H 8M5 Canada; 4 Knowledge Translation Program, Li Ka Shing Knowledge Institute, St Michael’s Hospital, Toronto, ON M5B 1W8 Canada; 5 Epidemiology Division, Dalla Lana School of Public Health, University of Toronto, Toronto, ON M5T 3M7 Canada

**Keywords:** Registration, Reporting, Systematic reviews, Methodology, Quality

## Abstract

**Background:**

The International Prospective Register of Systematic Reviews (PROSPERO) was launched in February 2011 to increase transparency of systematic reviews (SRs). There have been few investigations of the content and use of the database. We aimed to investigate the number of PROSPERO registrations from inception to 2017, and website usage in the last year. We also aimed to explore the epidemiological characteristics of and completeness of primary outcome pre-specification in a sample of PROSPERO records from 2017.

**Methods:**

The PROSPERO database managers provided us with data on the annual and cumulative number of SR registrations up to October 10, 2017, and the number of visits to the PROSPERO website over the year preceding October 10, 2017. One author collected data on the focus of the SR (e.g. therapeutic, diagnostic), health area addressed, funding source and completeness of outcome pre-specification in a random sample of 150 records of SRs registered in PROSPERO between April 1, 2017 and September 30, 2017.

**Results:**

As of October 10, 2017, there were 26,535 SRs registered in PROSPERO; guided by current monthly submission rates, we anticipate this figure will reach over 30,000 by the end of 2017. There has been a 10-fold increase in registrations, from 63 SRs per month in 2012 to 800 per month in 2017. In the year preceding October 10, 2017, the PROSPERO website received more than 1.75 million page views. In the random sample of 150 registered SRs, the majority were focused on a therapeutic question (78/150 [52%]), while only a few focused on a diagnostic/prognostic question (11/150 [7%]). The 150 registered SRs addressed 18 different health areas. Any information about the primary outcome other than the domain (e.g. timing, effect measures) was not pre-specified in 44/150 records (29%).

**Conclusions:**

Registration of SRs in PROSPERO increased rapidly between 2011 and 2017, thus benefiting users of health evidence who want to know about ongoing SRs. Further work is needed to explore how closely published SRs adhere to the planned methods, whether greater pre-specification of outcomes prevents selective inclusion and reporting of study results, and whether registered SRs address necessary questions.

## Background

Prior to 2011, users of health care evidence would be hard-pressed to find information about ongoing systematic reviews (SRs). Only a few organisations, including Cochrane and the Joanna Briggs Institute, disseminated protocols for SRs that were underway. Yet these organisations produce a minority of all published SRs [1, 2], so most would only become known at the time the SR was completed and published. This is despite the many benefits of registering SRs prior to their conduct. For example, prospective SR registration stimulates authors to anticipate methodological challenges that may arise, helps minimise potential for reporting bias by encouraging registrants to publish their SR and report all pre-specified outcomes and serves to reduce waste from unintended duplication of SRs by different teams of authors [3, 4]. Prospective SR registration is one of several processes that can facilitate optimal transparency, reproducibility and usability of SRs [5].

Calls for more extensive prospective registration of SRs started to gain traction around the late 2000s. An international group of epidemiologists, clinicians, statisticians and editors recommended in the 2009 Preferred Reporting Items for Systematic reviews and Meta-Analyses (PRISMA) statement that systematic reviewers provide registration information, including a registration number, for their SR [6, 7]. That same year, Tricco et al. advocated the establishment of a database to register SRs at inception, after identifying in an international survey of 348 authors that 199 SRs they had conducted had not been published [8]. And concerns about potential for reporting bias in unregistered SRs were raised after a 2010 study identified discrepancies between the pre-specified and reported outcomes of more than a fifth of Cochrane SRs (e.g. some outcomes were omitted, others were downgraded from primary to secondary) [9].

The world’s first international prospective register of systematic reviews (PROSPERO) was launched in February 2011 to mitigate these problems with SR transparency [10, 11]. PROSPERO is produced by the Centre for Reviews and Dissemination at the University of York (UK), and funded by the UK National Institute for Health Research (NIHR). The PROSPERO register accepts any SR with a health-related outcome, regardless of whether the focus is on a diagnostic, prognostic, genetic association or intervention question. Since November 2013, new protocols for Cochrane intervention and diagnostic test accuracy SRs have been added to PROSPERO. While SRs including any type of study design (e.g. randomised trials, cohort studies, qualitative studies) are eligible for inclusion, PROSPERO currently does not accept scoping reviews or literature reviews. For a SR on a methodological topic to be included, at least one patient-oriented or clinically relevant outcome needs to be included (therefore, methodological SRs looking only at, say, the reporting of particular methods in studies would not be included). The register requires systematic reviewers to provide information on 22 mandatory and 18 optional items, which were selected following an international consultation exercise [12]. Items include administrative information (e.g. SR title, anticipated or actual start date), SR methods (e.g. eligibility criteria, methods for collecting, handling and analysing data) and other general information (e.g. reference or URL for an accompanying SR protocol). PROSPERO also provides users with information on the status of a SR, with options including “ongoing”, “completed but not published” and “completed and published”. Once the SR is complete, authors can update the PROSPERO record to provide the full citation for the final report or publication of the SR, including the URL where available.

Along with facilitating SR transparency, the PROSPERO database is a valuable source of data for meta-research (i.e. research on research). For example, Booth et al. evaluated the number of registrations and number of visitors to the website between February 2011 and February 2012 [13]. Tricco et al. investigated discrepant outcome reporting between the PROSPERO record and publication of 98 SRs published before November 2013 [14]. Borah et al. used the start dates recorded in PROSPERO to estimate the time required to complete 195 registered SRs that were published before July 2014 [15]. And Sideri et al. evaluated how frequently SRs of orthodontic research published between 2012 and 2016 were registered a priori in PROSPERO [16]. To our knowledge, there has been no investigation of the number of registrations and usage of the PROSPERO website since its first year of operation. Also, epidemiological characteristics (e.g. health areas addressed, funding source) of SR registrations have not been examined. Further, there has been no evaluation of how completely outcomes are pre-specified in PROSPERO records. Complete pre-specification of SR outcomes is necessary to protect against bias due to selective inclusion and reporting of results, where the selection of data to include from studies, and subsequent reporting of results, is influenced by the nature of the findings [17–19].

We aimed to investigate the number of PROSPERO registrations from inception to 2017, and website usage in the last 12 months. We also aimed to explore the epidemiological characteristics of and completeness of primary outcome pre-specification in a 2017 sample of PROSPERO records.

## Methods

### Evaluation of the number of PROSPERO registrations and website usage

We obtained the following aggregate data upon request from the PROSPERO database managers:

- Total number of registrations by October 10, 2017;
- Annual registration numbers between February 1, 2011 and October 10, 2017;
- Country of the corresponding author for all registrations up to October 10, 2017;
- Number of website visits and page views of the PROSPERO website (https://www.crd.york.ac.uk/prospero/), and countries of users accessing the website, within the last 12 months (October 10, 2016 to October 10, 2017) and in the 12 months prior (October 10, 2015 to October 9, 2016). A website visit is counted any time a visitor reaches the PROSPERO website from somewhere outside the website domain. A page view is counted when a page on the PROSPERO website is loaded by a browser.

### Evaluation of epidemiological characteristics of and completeness of outcome pre-specification in PROSPERO records

We collected data on the focus of the SR (e.g. therapeutic, diagnostic), health area addressed, country of corresponding author, funding and completeness of outcome pre-specification in a random sample of 150 SRs registered in PROSPERO within the last 6 months. One author (MJP) downloaded the URLs of all records registered between April 1, 2017 and September 30, 2017 (*n* = 6070) from the PROSPERO database. The same author drew a random sample using the random number generator in Microsoft Excel, and retrieved the full PROSPERO record for all 150 SRs selected.

One author (MJP) collected data on epidemiologic characteristics from each PROSPERO record using a standardised data collection form, adopting the same terms used in a previous study evaluating the epidemiological characteristics of published SRs [1]. The focus of the SR was sought from the “Review question” field of the PROSPERO record, and classified as therapeutic (i.e. effects of a treatment/preventive intervention), epidemiologic (i.e. prevalence/incidence, or association between exposure and outcome), diagnostic (i.e. diagnostic test accuracy), prognostic (i.e. prognostic factors, biomarkers or clinical prediction rules) or other (e.g. qualitative analysis, measurement properties of instruments). Health area addressed was sought from the “Condition or domain being studied” field of the PROSPERO record, and classified using the International Classification of Diseases, Tenth Revision (ICD-10, http://apps.who.int/classifications/icd10/browse/2016/en). Country of corresponding author was sought from the “Country” and “Contact details for further information” fields of the PROSPERO record. Funding was sought from the “Funding sources/sponsors” field of the PROSPERO record, and classified as non-profit (e.g. government, university/hospital/research institute, charitable foundation), for-profit (e.g. pharmaceutical company) or no funding (as stated by the authors).

One author (MJP) classified completeness of outcome pre-specification for the primary outcome of the SR using the five-element framework developed by Saldanha et al. [20]. This framework recommends that authors of SRs pre-specify the:

1. Domain or outcome title (e.g. pain);
2. Specific measurement or technique/instrument used to make the measurement (e.g. 10-point visual analogue scale);
3. Specific metric or format of the outcome data from each participant that will be used for analysis (e.g. change in pain from baseline);
4. Method of aggregation or how data from each group will be summarised (e.g. mean change in pain from baseline);
5. Time points that will be used for analysis (e.g. at or closest to 6 weeks post-randomisation).

Information on outcome pre-specification was sought from the “Primary Outcome” field of the PROSPERO record and, if filled in, the “Timing and effect measures” field (the latter is an optional field on the PROSPERO registration form). Information was also sought from the “Data extraction (selection and coding)” and “Strategy for data synthesis” sections of the record. If the authors pre-specified multiple primary outcomes, we analysed only the outcome listed first in the “Primary Outcome” field of the PROSPERO record.

We performed all analyses using Stata version 14 software [21]. Data for all variables were summarised as frequency and percentage. We explored whether completeness of outcome pre-specification was associated with whether the focus of the SR was therapeutic or not. Associations were quantified as risk ratios, with 95% confidence intervals (CIs), using a log-binomial regression model.

## Results

### Registration statistics

**Fig. 1:**
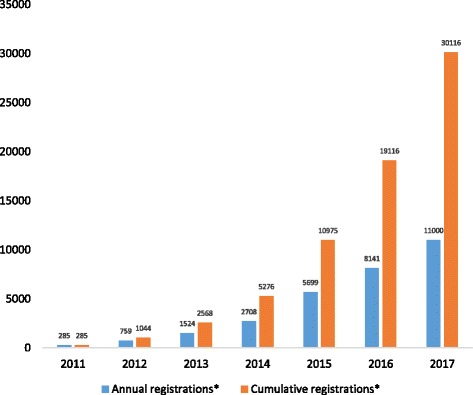
Annual and cumulative total number of PROSPERO registrations, 2011–2017. *Projected figures for year-end 2017 based on current monthly submission rates

As of October 10, 2017, there were 26,535 SRs registered in PROSPERO; guided by current monthly submission rates, we anticipate this figure will reach over 30,000 by the end of 2017. There has been a 10-fold increase in registrations, from 63 SRs per month in 2012 (the first complete calendar year of registrations) to 800 per month in 2017 (Fig. 1).

PROSPERO includes registrations from all over the world, with more than 100 countries contributing. The highest number of registrations originate from England, who were responsible for 4828/26,535 (18%) records registered from inception to October 10, 2017 (Table 1).

**Table 1:** Number of records registered in PROSPERO by the top 10 contributing countries, from inception to October 10, 2017

Country	Number (%) registrations
England	4828 (18%)
Australia	2813 (11%)
USA	2530 (10%)
China	2367 (9%)
Brazil	2342 (9%)
Canada	2187 (8%)
The Netherlands	872 (3%)
Germany	665 (3%)
Scotland	629 (2%)
Italy	564 (2%)

The top 10 countries are responsible for 75% of all registrations. Only the country of the corresponding author is considered

### Website usage

In the year preceding October 10, 2017, the PROSPERO website received more than 1.75 million page views. Visits to the website almost doubled in the last 12 months, with 525,750 visits occurring between October 2016 and October 2017 compared to 327,439 visits from October 2015 to October 2016. Use of PROSPERO is global, with the greatest number of website visits in the past year originating from the UK (117,878 visits).

### Epidemiological characteristics of SRs registered in PROSPERO

In the random sample of 150 SRs registered between April 1, 2017 and September 30, 2017, the majority were focused on a therapeutic question (78/150 [52%]; Table 2). Almost a third of registrations addressed an epidemiologic question (47/150 [31%]), while only a few focused on a diagnostic/prognostic question (11/150 [7%]). The 150 SRs addressed a wide range of health areas; 18 ICD-10 chapters were recorded across the records. The top three most common ICD-10 chapters were diseases of the circulatory system (16/150 [11%]), mental and behavioural disorders (16/150 [11%]) and diseases of the musculoskeletal system and connective tissue (15/150 [10%]). Three ICD-10 chapters—diseases of the eye and adnexa, diseases of the ear and mastoid process and external causes of morbidity and mortality—were not covered by a single SR. Corresponding authors came from 28 countries, with England (27/150 [18%]), Australia (21/150 [14%]) and China (17/150 [11%]) the most common. Almost half of the SRs were funded by a non-profit source (66/150 [44%]), while the remainder were declared as being conducted without any funding. None of the SRs were funded by a for-profit source. Also, all of the registrations were for non-Cochrane SRs.

**Table 2:** Epidemiological characteristics of a random sample of 150 SRs registered in PROSPERO between April 1, 2017 and September 30, 2017

Characteristic	Frequency (%)
Focus of SR	
Therapeutic	78 (52%)
Epidemiologic	47 (31%)
Diagnostic	7 (5%)
Prognostic	4 (3%)
Other	14 (9%)
Health area addressed (ICD-10 chapter)	
Diseases of the circulatory system	16 (11%)
Mental and behavioural disorders	16 (11%)
Diseases of the musculoskeletal system and connective tissue	15 (10%)
Factors influencing health status and contact with health services	12 (8%)
Symptoms, signs and abnormal clinical and laboratory findings, not elsewhere classified	11 (7%)
Certain infectious and parasitic diseases	10 (7%)
Neoplasms	10 (7%)
Diseases of the digestive system	9 (6%)
Diseases of the genitourinary system	8 (5%)
Endocrine, nutritional and metabolic diseases	8 (5%)
Diseases of the respiratory system	7 (5%)
Diseases of the nervous system	6 (4%)
Pregnancy, childbirth and the puerperium	6 (4%)
Injury, poisoning and certain other consequences of external causes	4 (3%)
Certain conditions originating in the perinatal period	3 (2%)
Congenital malformations, deformations and chromosomal abnormalities	3 (2%)
Diseases of the blood and blood-forming organs and certain disorders involving the immune mechanism	3 (2%)
Diseases of the skin and subcutaneous tissue	3 (2%)
Diseases of the eye and adnexa	0 (0%)
Diseases of the ear and mastoid process	0 (0%)
External causes of morbidity and mortality	0 (0%)
Country of corresponding author	
England	27 (18%)
Australia	21 (14%)
China	17 (11%)
Brazil	16 (11%)
USA	16 (11%)
Canada	12 (8%)
Other (fewer than 5 reviews per country, including Argentina, Bangladesh, Belgium, Denmark, Ethiopia, France, Germany, Greece, Hong Kong, Hungary, India, Iran, Ireland, Italy, Japan, New Zealand, Norway, Scotland, Spain, Sweden Switzerland, The Netherlands)	41 (27%)
Funding of SR	
Non-profit	66 (44%)
For-profit	0 (0%)
Authors specified there was no funding	84 (56%)

*ICD-10* International Classification of Diseases, Tenth Revision, *SR* systematic review

### Completeness of primary outcome pre-specification in PROSPERO records

Pre-specification of primary outcomes was incomplete in most of the 150 SRs registered between April 1, 2017 and September 30, 2017 (Table 3). The primary outcome was completely pre-specified (i.e. all five components were stated) in the PROSPERO record in 9/150 (6%) cases. In 44/150 (29%) cases, the domain was the only information about the primary outcome that was pre-specified. Approximately 40% of the PROSPERO records included information about the specific measurement, metric or method of aggregation for the primary outcome. Fewer PROSPERO records (25/150 [17%]) included information on the time points of interest for the primary outcome of the SR. Completeness of pre-specification was similar between therapeutic SRs and non-therapeutic SRs for all outcome components, except for time point, which was pre-specified more often in therapeutic SRs (20/78 [26%] therapeutic SRs versus 5/72 [7%] non-therapeutic SRs; risk ratio 3.69, 95% CI 1.46 to 9.32).

**Table 3:** Completeness of primary outcome pre-specification in a random sample of 150 SRs registered in PROSPERO between April 1, 2017 and September 30, 2017

Components of outcome	Number (%) all SRs	Number (%) therapeutic SRs	Number (%) non-therapeutic SRs	Risk ratio (95% CI)
	*n* = 150	*n* = 78	*n* = 72	
Domain	150 (100%)	78 (100%)	72 (100%)	NA
Specific measurement	65 (43%)	35 (45%)	30 (42%)	1.08 (0.75, 1.56)
Specific metric	63 (42%)	31 (40%)	32 (44%)	0.89 (0.61, 1.30)
Method of aggregation	69 (46%)	36 (46%)	33 (46%)	1.01 (0.71, 1.42)
Time point	25 (17%)	20 (26%)	5 (7%)	3.69 (1.46, 9.32)
Only domain specified	44 (29%)	21 (27%)	23 (32%)	0.84 (0.51, 1.39)
Outcome completely pre-specified	9 (6%)	7 (9%)	2 (3%)	3.23 (0.69, 15.04)

*CI* confidence interval, *NA* not applicable, *SR* systematic review

## Discussion

Registration of SRs in PROSPERO has increased rapidly, from 285 records in the year of inception (i.e. 2011) to 30,000 by the end of 2017. Half a million visits to the website occurred in the last 12 months from all over the world, most commonly from the UK. In a random sample of 150 SRs registered in PROSPERO between April 1, 2017 and September 30, 2017, the majority were focused on a therapeutic or epidemiologic question, and are being conducted without a dedicated funding source. A wide range of health areas were addressed across the 150 SRs, the most common being diseases of the circulatory system, mental and behavioural disorders and diseases of the musculoskeletal system and connective tissue. Pre-specification of primary outcomes was incomplete in most of the 150 records, with almost a third specifying the domain only.

### Strengths and limitations

A strength of our study is that the PROSPERO database managers provided us with routinely collected data on the number of PROSPERO registrations and website usage, which removed the potential for errors due to manual data collection. Further, we were able to explore trends in these variables over time, unlike a previous evaluation which was limited to the first year of activity [13]. However, there are also some limitations. We analysed other variables (epidemiological characteristics and completeness of outcome pre-specification) in a random sample of SRs registered in PROSPERO between April and September 2017 only. Therefore, our frequency statistics may not generalise to SRs registered earlier. It is possible that some errors exist in our data on epidemiological characteristics and completeness of outcome pre-specification, given that data collection and classification were performed by one author only. However, we expect the number of errors to be low given the extensive experience that the data collector (MJP) has from recording this type of information in previous studies [1, 14, 17, 22].

### Comparison with other studies

Compared with a previous analysis of the characteristics of PROSPERO registrations [13], the geographical scope of SRs registered has changed considerably over time. Registrations in the first year (2011–2012) came from 33 different countries, whereas that figure has risen to over 100 countries now. England remains the country responsible for most registrations, and Italy has moved into the list of top ten contributors, overtaking Denmark. The number of registrations submitted by authors based in Australia and China has also increased; in the first year of operation, these countries were in fourth and eighth position on the list of top contributing countries [13], and have moved into second and fourth position, respectively.

### Explanations and implications

The exponential increase in SR registrations from 2011 to 2017 is a surprising, albeit welcome, development. Unlike for clinical trialists, who rapidly embraced trial registration only after the International Committee of Medical Journal Editors (ICMJE) announced that they would no longer publish trials that were not registered at inception [23], SR registration is not yet required by most journals (nor is it an ICMJE journal requirement). Therefore, the motivation of systematic reviewers to register their SR is likely due to other factors. It is possible that authors are paying attention to the increasing number of publications promoting the advantages of pre-registration of scientific studies and open science practices [24–26]. The uptake of registration could also be driven in part by journals that endorse the PRISMA Statement, which encourages SR registration in item 5 [6]. In addition, awareness of the benefits of SR registration likely increased following the dissemination of the PRISMA for Protocols Statement in January 2015 [27, 28], which mentions PROSPERO specifically in item 2. Further, many authors of SRs may be trialists as well, so an acceptance of the need for trial registration may have translated to an acceptance of SR registration.

The epidemiological characteristics of the registered SRs we examined share some similarities, and some differences, with a recent sample of completed SRs [1]. In both samples, most of the SRs focused on a therapeutic question (55% of 300 SRs indexed in MEDLINE® in February 2014 versus 52% of 150 SRs registered in PROSPERO in 2017), and few focused on a diagnostic/prognostic question (11 versus 7%, respectively). This possibly reflects the fact that methods for therapeutic SRs are more established than methods for other types of SRs [29, 30]. Diseases of the circulatory system was one of the most common health areas addressed in both samples; a positive sign given that conditions such as ischaemic heart disease and stroke are leading causes of mortality globally [31, 32]. Neoplasms and certain infectious and parasitic diseases—other leading causes of mortality [32]—were the most common areas addressed in the SRs indexed in MEDLINE® in February 2014 (in 16 and 14% of 300 SRs, respectively), yet both diseases were addressed in fewer (7%) of the 150 SRs registered in PROSPERO in 2017. We encourage systematic reviewers planning SRs to align their topic with global burden of disease data to ensure they are targeting clinically important questions [33].

None of the 150 registered SRs that we studied in detail were funded by a for-profit source. This is concerning given that other research has revealed a proliferation of meta-analyses are being commissioned by industry, most of which are not registered or published [34]. Clearly, more work is needed to encourage for-profit companies to embrace SR transparency practices. In the future, it would be worthwhile to explore (e.g. via surveys and interviews) the reasons for non-registration of SRs by investigators working in for-profit and non-profit organisations.

The incomplete pre-specification of outcomes that we observed in PROSPERO records is consistent with that observed in previous studies evaluating outcomes in Cochrane SR protocols [17, 18] and published SRs [17, 20]. This is likely because full pre-specification of outcomes is not yet a mandatory requirement for SR registration. We believe this should be reconsidered. There is emerging evidence that multiple results are often available for the same outcome in clinical trials (e.g. pain is measured using three scales, each at two time points), and that failure to completely pre-specify SR outcomes can lead to challenges with selecting and interpreting results from the included trials [17, 35, 36]. For example, Mayo-Wilson et al. found that across 21 trials of gabapentin for neuropathic pain, the number of unique within-trial results for pain intensity that systematic reviewers could select from ranged from 1 to 68 (because of within-trial multiplicity of outcome measurements, metrics, methods of aggregation and time points). For this reason, the total number of possible meta-analyses for pain intensity that systematic reviewers could (in theory) calculate was more than 34 trillion [36]. To deal with multiplicity challenges such as these, and prevent cherry-picking of the most favourable results when multiple are available within studies, systematic reviewers should make greater use of the “Timing and effect measures” field in PROSPERO, to completely pre-specify the outcomes of interest to the SR. In addition, such information in PROSPERO records should be accurately reflected in public versions of SR protocols and vice versa, and any amendments should be documented in completed SR articles.

There are several avenues of further work relating to PROSPERO. The field of knowledge synthesis is evolving to include other types of reviews, such as scoping reviews [37], living SRs [38] and rapid reviews [39]. Given the rapidly changing environment and methods, PROSPERO may need to adapt the criteria for inclusion in the database, as well as items on the registration form, to reflect the current state of the field. Also, it would be worthwhile to conduct a large-scale investigation of publication rates of registered SRs, the prevalence of and reasons for discontinued SRs and discrepancies in the planned and reported SR methods. Finally, that 30,000 SRs are underway suggest that the mass production of SRs previously discussed [40, 41] shows no sign of waning. It remains to be seen whether all of these SRs address questions that are relevant to end-users (e.g. patients, health care providers and policy makers), are well conducted, and are free of financial conflicts of interest. The PROSPERO management team have advised us that to minimise the potential for redundancy of SRs, forthcoming changes to the user interface will require those registering SRs to consider whether similar SRs already exist, and whether a new SR is necessary (L. Stewart, personal communication).

## Conclusion

Registration of SRs in PROSPERO increased rapidly between 2011 and 2017, thus benefiting users of health evidence who want to know about ongoing SRs. Further work is needed to explore how closely published SRs adhere to the planned methods, whether greater pre-specification of outcomes prevents selective inclusion and reporting of study results, and whether registered SRs are addressing necessary questions.

## Abbreviations

CI: Confidence interval
ICD-10: International Classification of Diseases, Tenth Revision
NIHR: National Institute for Health Research
PRISMA: Preferred Reporting Items for Systematic reviews and Meta-Analyses
SR: Systematic review
